# *Ziziphus spina-christi* Leaf Extract Suppressed Mercury Chloride-Induced Nephrotoxicity via Nrf2-Antioxidant Pathway Activation and Inhibition of Inflammatory and Apoptotic Signaling

**DOI:** 10.1155/2019/5634685

**Published:** 2019-11-13

**Authors:** Rafa S. Almeer, Gadah Albasher, Fatimah Alotibi, Saud Alarifi, Daoud Ali, Saad Alkahtani

**Affiliations:** ^1^King Saud University, College of Science, Department of Zoology, Riyadh, Saudi Arabia; ^2^King Saud University, College of Science, Department of Botany and Microbiology, Riyadh, Saudi Arabia

## Abstract

Exposure to heavy metals, including mercury chloride (HgCl_2_), is associated with severe health problems. This study was designed to investigate HgCl_2_-induced nephrotoxicity and evaluate the protective role of *Ziziphus spina-christi* leaf extract (ZSCLE). Four randomly selected groups containing seven rats were used. For a period of 28 days, the control group was administered 0.9% saline solution; the second group was administered 300 mg/kg ZSCLE; the third group was administered 0.4 mg/kg HgCl_2_ dissolved in 0.9% physiological saline solution; and the fourth group was administered an oral supplement of 300 mg/kg ZSCLE one hour after HgCl_2_ administration. HgCl_2_ intoxication resulted in Hg accumulation in renal tissue; decreases in body weight, kidney index, and glutathione content and superoxide dismutase, catalase, glutathione peroxidase, and glutathione reductase activities; increases in creatinine, urea, Kim-1 expression, lipid peroxidation, and nitric oxide production; suppression of the Nrf2-antioxidant response pathway; upregulation of *Il1β*, *Tnfα*, and *Nos2*; and potentiation of proapoptotic activity. ZSCLE exerted beneficial effects against mercury-induced renal toxicity and significantly reversed these alterations to near normal values. These effects resulted from its chelation and antioxidant, anti-inflammatory, and antiapoptotic activities. ZSCLE may prevent or minimize the pathological changes induced by mercury in the kidney.

## 1. Introduction

Mercury (Hg) is classified among the most dangerous environmental hazards. Hg is dispersed widely in the environment in metallic, organic, and inorganic forms owing to natural or human activities, including mining, industrial and municipal wastewater discharge, agriculture, and incineration [1]. Moreover, Hg is used in various industries, for example, in batteries, cosmetics, dental amalgam, and thermometers [2]. Humans are exposed to Hg in air, water, and contaminated food, particularly seafood; the average biological half-life may be extended to 60 days [3]. Exposure to Hg at high or even at low doses causes deleterious effects, such as vomiting, nausea, diarrhea, pulmonary damage, hypertension, neurotoxicity, reproductive dysfunctions, and nephrotoxicity [3]. Inorganic mercury accumulates mainly in the kidneys, resulting in acute renal damage [3]. The effect of Hg on renal function can be evaluated through the estimation of the glomerular and tubular function. Levels of serum creatinine and blood urea nitrogen are also used as a physiological indicator for Hg-induced nephrotoxicity in humans [4].

The exact mechanisms of Hg-induced nephrotoxicity are still unclear. However, numerous factors have been suggested to play a fundamental role in Hg intoxication [5]. Mercury has a strong affinity to sulfhydryl group-containing molecules, such as glutathione, which results in the disturbance of the cellular antioxidant system and the development of oxidative stress in renal tissue [3]. Mercury also binds competitively with essential elements, such as zinc and selenium, leading to their elimination and disturbing several physiological and regulatory functions [6]. Inflammation and apoptosis are also reported to participate in Hg toxicity [7, 8].

The use of antioxidants has been suggested to alleviate heavy metal intoxication with minimal side effects in various experimental models [9, 10]. *Ziziphus spina-christi* is an edible plant, known also as Christ's thorn, Jujube, Nabka, and Sidr. *Z. spina-christi* is a species in the family Rhamnaceae and grows mainly in hot and dry regions [11]. The seeds comprise 18.6% protein and 28.5% lipids, whereas the flesh comprises 80.6% carbohydrates. The leaves are rich in calcium and magnesium, and a high ascorbic acid content has been recorded in the mesocarp [11]. Previous reports showed that *Ziziphus spina-christi* leaf extract (ZSCLE) contains numerous phytochemical constituents, such as flavonoids, alkaloids, tannins, triterpenoid, phytosterols, saponins, and essential oils [12]. Several medicinal characteristics of the genus *Ziziphus* have been recorded, including antioxidant, anti-inflammatory, hepatoprotective, hypoglycemic, antitumor, hypotensive, antidiarrheal, antimalarial, antiplasmodium, and immunomodulatory activities [13]. To the best of our knowledge, the potential protective effect of *Z. spina-christi* against mercury-induced nephrotoxicity has not yet been explored. Therefore, the aim of the current study was to determine whether *Z. spina-christi* was able to rescue renal tissue from the effects of mercury intoxication through the evaluation of mercury concentration, kidney function parameters, redox homeostasis, inflammatory response, and expression of apoptotic proteins in the renal tissue of rats.

## 2. Materials and Methods

### 2.1. Preparation of *Z. spina-christi* Leaf Extract

*Z. spina-christi* leaves were collected from a public garden in the east of Riyadh, Saudi Arabia. The leaves were identified by an expert taxonomist from the Botany Department, College of Science, Riyadh, Saudi Arabia*. Z. spina-christi leaves* were cleaned of dust under running tap water and then air-dried in the shade. The dried ZSC leaves were finely powdered and immersed in 80% (*v*/*v*) methanol at 4°C for 72 h. The extract was filtered, and the supernatant was evaporated under reduced pressure to a semidry state using a rotary evaporator at 45°C and then dissolved in distilled water. The obtained extract was designated as *Z. spina-christi* leaf extract (ZSCLE) and stored at -20°C until further analysis.

### 2.2. Animals and Experimental Design

Twenty-eight adult male Wistar rats, 9–10 weeks of age and weighing 120–150 g, were obtained from the animal facility of College of Science, Riyadh, Saudi Arabia. The rats were housed in the Zoology Department, College of Science, Riyadh, Saudi Arabia, under standard laboratory conditions, with a 12 h light/dark cycle at a fixed temperature (22°C–25°C) and access to standard pelleted rodent feed and water *ad libitum*. To evaluate the protective effect of ZSCLE against mercury-induced nephrotoxicity in rats, the study tested four randomly selected groups (*n* = 7). The first and the second groups were the control and ZSCLE-treated groups, and the other two groups were exposed to mercury. Based on our previous study, the first group (control) was administered 0.9% saline solution for 28 days and the second group was administered with 300 mg/kg ZSCLE for 28 days. The third group was administered with 0.4 mg/kg HgCl_2_ (CAS Number 7487-94-7; Sigma-Aldrich, St. Louis, MO, USA) dissolved in 0.9% physiological saline solution for 28 days. This dose was previously reported to yield no observable signs of toxicity [14]. The fourth group was supplemented with an oral administration of 300 mg/kg ZSCLE one hour after the administration of HgCl_2_ for 28 days. The rats were sacrificed following an overdose of pentobarbital (100 mg/kg i.p.) 24 h after final treatment. The kidneys were quickly removed and homogenized in 10 mM phosphate buffer (pH 7.4). The homogenate was centrifuged for 10 min (3000 × *g*) at 4°C, and the supernatant was stored at -20°C for further investigation. To determine the kidney function, blood samples were also collected. The rules and guidelines governing the handling and care of animals were approved by the Department of Zoology, College of Science, Saudi Arabia Committee for Laboratory Animal Care and were in accordance with the National Institute of Health (NIH) Guidelines for the Care and Use of Laboratory Animals, 8th edition (NIH Publication No. 85-23 revised 1985).

### 2.3. Determination of Mercury Concentration in the Kidney

Mercury accumulation in the kidney was estimated using an atomic absorption spectrophotometer (Perkin Elmer 3100) as described previously by [15]. Briefly, 200 mg of kidney tissue was digested with 2 mL of concentrated nitric acid (HNO_3_) in an oven at 100°C for 6 h. After digestion, the sample volume was completed into 25 mL with deionized distal H_2_O. Afterward, an appropriate volume of sample was injected into a graphite furnace at 253.7 nm. The samples were analyzed in duplicate, and the mercury concentration was determined from the standard curve on wet kidney tissue basis as *μ*g/g wet tissue.

### 2.4. Kidney Weight Estimation

The kidney weight was estimated using a sensitive weighing balance (Radwag, Model AS220/C/2, Clarkson Laboratory and Supply Inc., Chula Vista, CA, USA), from which the kidney index was determined using the following formula:

(1) $$\begin{array}{c} \text{Kidney index }\left(\text{KI}\right)=\frac{\text{Left kidney }\left(\text{LT}\right)}{\text{Body weight}}\times 100. \end{array}$$

### 2.5. Biochemical Analysis

#### 2.5.1. Serum Kidney Function Parameters

The serum urea and creatinine levels were determined to assess kidney function in accordance with the instruction manual provided by Randox Laboratories Ltd. (Crumlin, United Kingdom).

#### 2.5.2. Oxidant/Antioxidant Status Analysis

Lipid peroxidation (LPO) assessment was based on the quantity of formed malondialdehyde (MDA), a lipid peroxidation indicator, according to the method of Ohkawa et al. [16]. Nitric oxide (NO) was assessed using the Griess reagent [17]. The amount of reduced glutathione (GSH) in the kidney homogenates was determined utilizing the method described by Ellman [18]. The activity of superoxide dismutase (SOD) was determined according to the method of Nishikimi et al. [19]. Hydrogen peroxide decomposer enzyme (catalase (CAT)) activity was determined through the measurement of the decomposition rate of hydrogen peroxide (H_2_O_2_) at 240 nm, based on the method described by Aebi [20]. Finally, the activities of glutathione peroxidase (GPx) and glutathione reductase (GR) were assayed using the methods of Paglia and Valentine [21] and De Vega et al. [22], respectively.

### 2.6. Proinflammation Cytokine Biomarker Assay

Renal levels of tumor necrosis factor-*α* (TNF-*α*) and interleukin-1*β* (IL-1*β*) were measured using kits in accordance with the manufacturer's protocol.

### 2.7. Kidney Injury Molecule-1

Renal homogenate was used to assess Kim-1 using an ELISA kit (R&D Systems) in accordance with the manufacturer's protocol.

### 2.8. Real-Time PCR

Total RNA from kidney tissue was extracted using the TRIzol Reagent and was then converted to cDNA using RevertAid™ H Minus Reverse Transcriptase (Fermentas, Thermo Fisher Scientific, Canada) in accordance with the manufacturer's instructions. For gene expression analysis, quantitative real-time PCR was employed using a QuantiFast SYBR Green RT-PCR kit (Qiagen, Hilden, Germany). Sense and antisense primers were obtained from Jena Bioscience (Jena, Germany) and are listed in Table 1. All reactions were performed in duplicate by using a ViiA™ 7 System (Thermo Fisher Scientific, CA, USA). The PCR cycling conditions were set as follows: initial denaturation at 95°C for 12 min, followed by 40 cycles of denaturation at 94°C for 60 s, annealing at 55°C for 60 s, and extension at 72°C for 90 s, with a final extension at 72°C for 10 min. The relative differences in gene expression between different groups were determined by using the ^ΔΔ^Ct method [23]. Glyceraldehyde-3-phosphate dehydrogenase (*Gapdh*) was used as the reference gene.

### 2.9. Histological Procedures

Kidney tissues were fixed in 10% formaldehyde/PBS for 24 h. Renal tissues were dehydrated using high-grade alcohol, embedded in paraffin, and sliced into 4 to 5 *μ*m sections. The specimens were stained with hematoxylin and eosin. Finally, the slides were examined by using a Nikon Eclipse E200-LED (Tokyo, Japan) microscope at 400x magnification.

### 2.10. Statistical Analysis

The results were expressed as the mean ± standard error of the mean (SEM) of seven rats. The data were compared by one-way analysis of variance (ANOVA). Duncan's post hoc multiple tests were performed. *P* values of <0.05 were considered statistically significant.

## 3. Results

**Figure 1:**
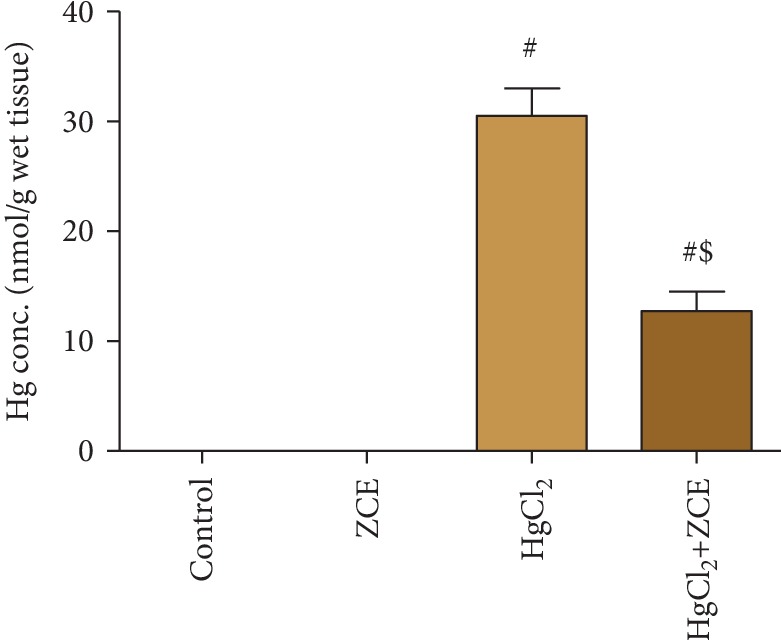
The bioaccumulation of mercury in renal tissue following treatment with *Ziziphus spina-christi* leaf extract (ZSCLE) and/or mercury chloride (HgCl_2_) for 28 days. The obtained results were expressed as the mean ± SD (*n* = 7). ^#^*P* < 0.05 indicates significance compared with the control animals; ^$^*P* < 0.05 indicates significance compared with HgCl_2_-treated animals.

**Figure 2:**
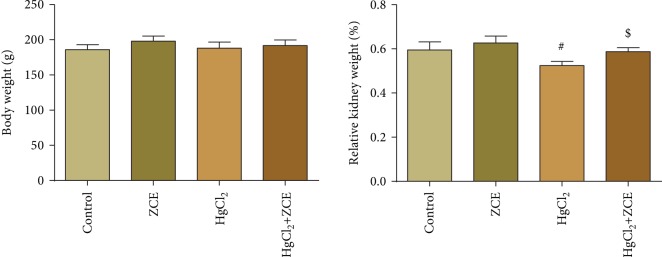
Determination of the body weight and kidney index following treatment with *Ziziphus spina-christi* leaf extract (ZSCLE) and/or mercury chloride (HgCl_2_) for 28 days. The obtained results were expressed as the mean ± SD (*n* = 7). ^#^*P* < 0.05 indicates significance compared with the control animals; ^$^*P* < 0.05 indicates significance compared with HgCl_2_-treated animals.

**Figure 3:**
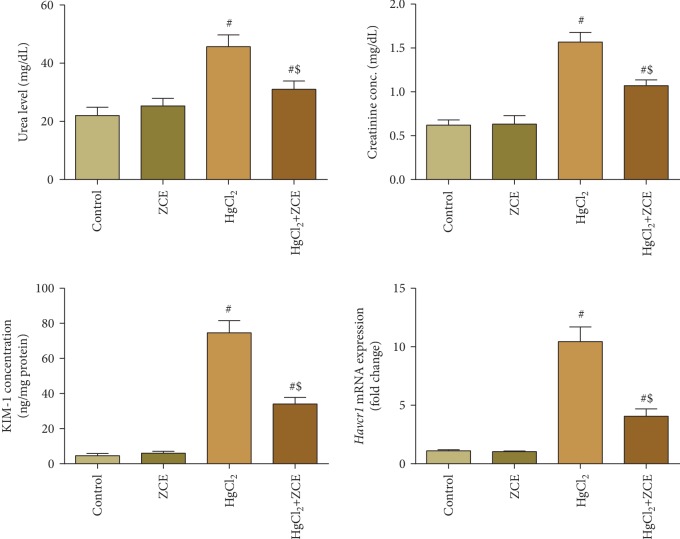
Illustration of the kidney function indices, including creatinine, urea, and Kim-1 expression, following treatment with *Ziziphus spina-christi* leaf extract (ZSCLE) and/or mercury chloride (HgCl_2_) for 28 days. The obtained results were expressed as the mean ± SD (*n* = 7). ^#^*P* < 0.05 indicates significance compared with the control animals; ^$^*P* < 0.05 indicates significance compared with HgCl_2_-treated animals. The mRNA expression data for *Havcr1* (*Kim1*) is presented as the mean ± SD of triplicate assays normalized to the expression of *Gapdh* and expressed as fold change on a log2 scale for the control and HgCl_2_-treated animals.

Rats exposed to HgCl_2_ showed a significant elevation (*P* < 0.05, 32.33%) in Hg bioaccumulation in renal tissue compared with the control group (Figure 1). The oral administration of HgCl_2_ for 28 days induced a significant reduction in the kidney index (-11.17%) compared with the control group (Figure 2). Furthermore, Hg bioaccumulation in the renal tissue was concomitant with kidney dysfunction, as indicated by the significant elevation in the serum levels of creatinine (152.68%), urea (107.57%), and Kim-1 (1498.85%) (Figure 3). However, ZSCLE supplementation abolished all the deleterious effects of Hg, as indicated by the significant decrease in Hg accumulation and amelioration of kidney function biomarkers, body weight, and kidney index compared with HgCl_2_-treated rats. Treatment with ZSCLE alone did not affect the kidney index or kidney function parameters.

**Figure 4:**
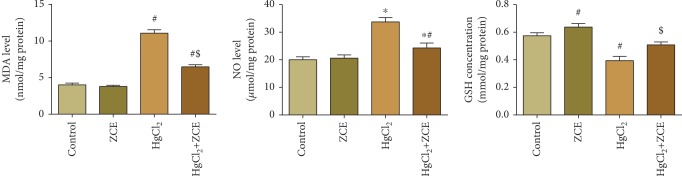
The effect of *Ziziphus spina-christi* leaf extract (ZSCLE) after the administration of mercury chloride (HgCl_2_) on renal lipid peroxidation (LPO), nitric oxide (NO), and glutathione (GSH). The obtained results were expressed as the mean ± SD (*n* = 7). ^#^*P* < 0.05 indicates significance compared with the control animals; ^$^*P* < 0.05 indicates significance compared with HgCl_2_-treated animals.

One-way ANOVA revealed that the administration of inorganic mercury to rats caused a drastic elevation (*P* < 0.05) in the lipid peroxidation (LPO) level and NO production (176.30% and 68.66%, respectively). Oxidative stress biomarkers were increased, coupled with a significant depletion in GSH content (-30.98%). ZSCLE posttreatment after HgCl_2_ administration abrogated the level of oxidative stress biomarkers and restored the levels to those of the control values (Figure 4).

**Figure 5:**
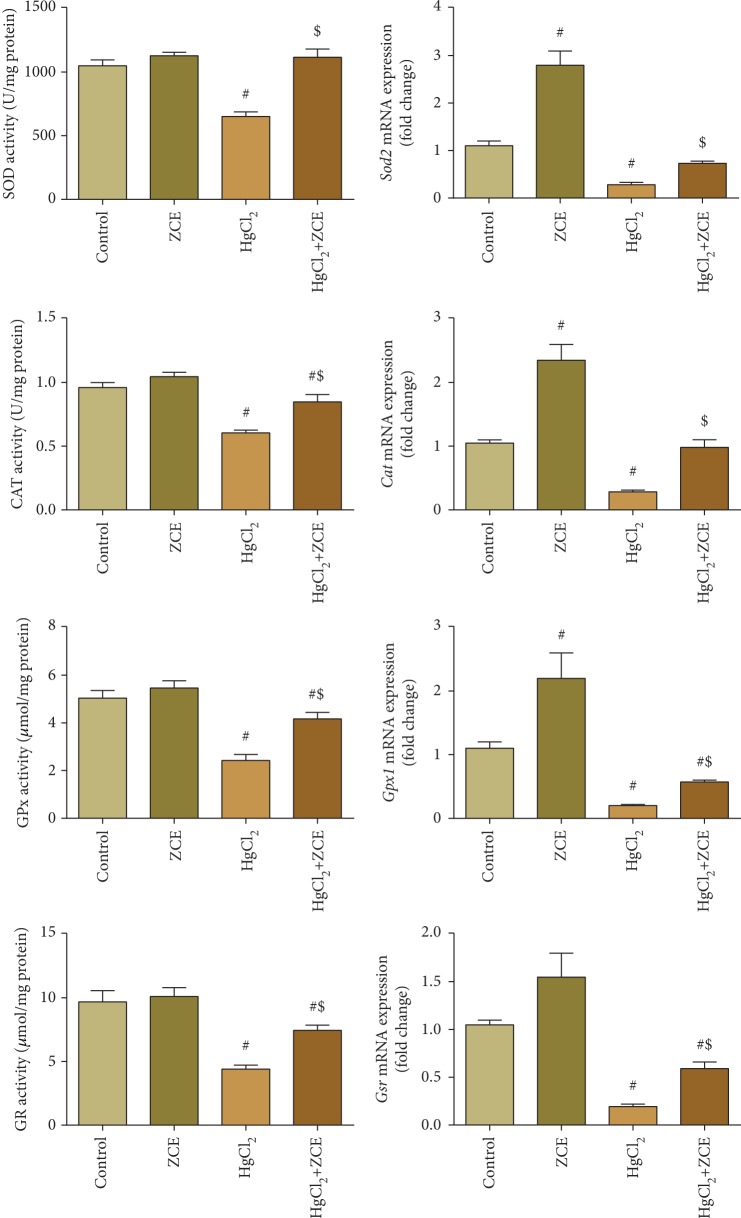
Treatment with *Ziziphus spina-christi* leaf extract (ZSCLE) activated superoxide dismutase (SOD), catalase (CAT), glutathione peroxidase (GPx), and glutathione reductase (GR), as well as their gene expression, and protected the kidneys against HgCl_2_-induced oxidative stress. The obtained biochemical results were expressed as the mean ± SD (*n* = 7). ^#^*P* < 0.05 indicates significance compared with the control animals; ^$^*P* < 0.05 indicates significance versus HgCl_2_-intoxicated animals. The mRNA expression data for *Sod2*, *Cat*, *Gpx1*, and *Gsr* are presented as the mean ± SD of triplicate assays normalized to the expression of *Gapdh* and expressed as fold change on a log2 scale for the control and HgCl_2_-treated animals.

Exposure to inorganic mercury induced significant inhibition (*P* < 0.05) of the antioxidant enzyme activities of superoxide dismutase (SOD, -37.96%), H_2_O_2_ decomposer enzyme (CAT, -37.08%), glutathione peroxidase (GPx, -51.90%), and glutathione reductase (GR, -54.54%), as shown in Figure 5. These alterations in the antioxidant enzyme were prevented by ZSCLE posttreatment after HgCl_2_ treatment. Consistent with the obtained biochemical results, qRT-PCR data revealed that mRNA expressions of *Sod2*, *Cat*, *Gpx1*, and *Gsr* were notably downregulated (fivefold change) in HgCl_2_-treated rats, and ZSCLE posttreatment prevented the alteration of these genes (Figure 5).

**Figure 6:**
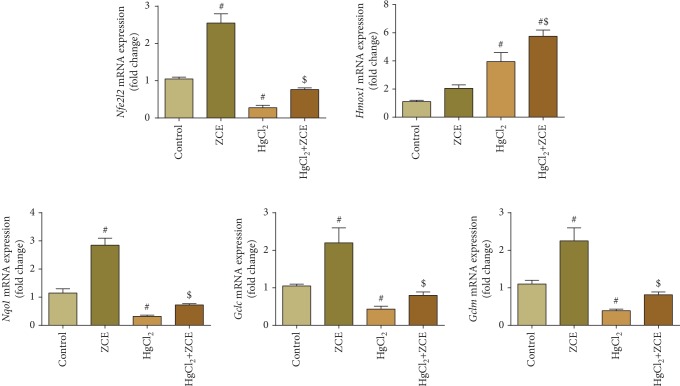
Treatment with *Ziziphus spina-christi* leaf extract (ZSCLE) activated nuclear factor (erythroid-derived 2)-like 2 (*Nfe2l2*), heme oxygenase-1 (*Hmox1*), NAD(P)H dehydrogenase [quinone] 1 (*Nqo1*), glutamate-cysteine ligase catalytic subunit (*Gclc*), and glutamate-cysteine ligase modifier subunit (*Gclm*) gene expression and protected renal tissue against HgCl_2_-induced oxidative damage. mRNA expression data were presented as the mean ± SD of triplicate assays normalized to the expression of *Gapdh* and expressed as fold change on a log2 scale for the control and HgCl_2_-treated animals.

To understand the antioxidant mechanism of ZSCLE in HgCl_2_-induced nephrotoxicity, the expression of the Nrf2-antioxidant response pathway in the kidneys of rats was investigated. Mercury exposure caused a significant downregulation in the mRNA expression of *Nfe2l2* (fivefold change), *Nqo1* (threefold change), *Gclc* (twofold change), and *Gclm* (twofold change); meanwhile, *Hmox1* was upregulated (twofold change) (Figure 6). However, ZSCLE posttreatment alleviated the deleterious effect of Hg. Collectively, the qRT-PCR findings indicated the protective effect of ZSCLE against nephrotoxicity mediated by Hg-induced oxidative injury (Figure 6).

**Figure 7:**
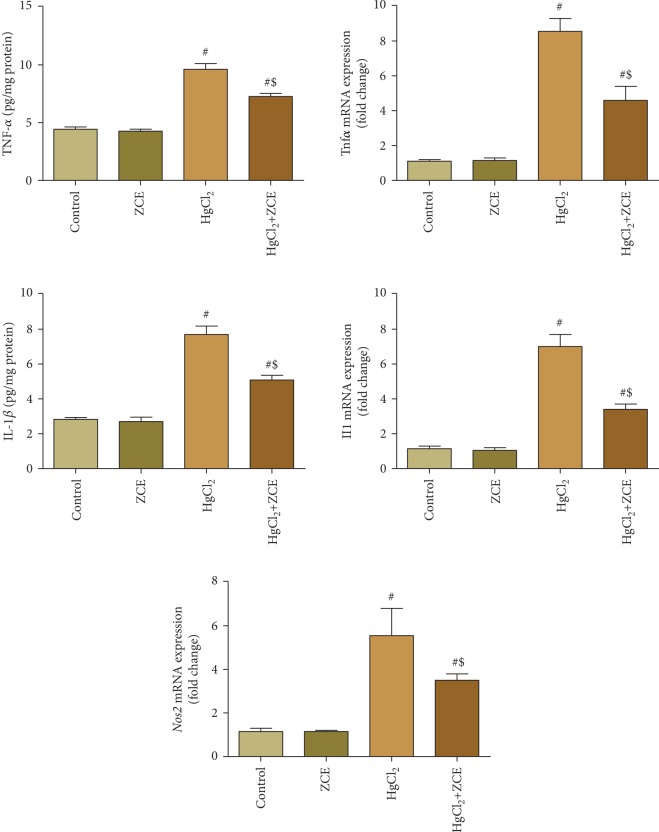
*Ziziphus spina-christi* leaf extract (ZSCLE) suppressed the expression of interleukin-1*β* (IL-1*β*), tumor necrosis factor-*α* (TNF-*α*), and inducible nitric oxide synthase (iNOS) expression following HgCl_2_ intoxication. The biochemical analyses of IL-1*β* and TNF-*α* were expressed as the mean ± SD (*n* = 7). ^#^*P* < 0.05 indicates significance compared with the control values; ^$^*P* < 0.05 indicates significance compared with HgCl_2_-treated animals. mRNA expression data for *Il1β*, *Tnfα*, and *Nos2* were presented as the mean ± SD of triplicate assays normalized to the expression of *Gapdh* and expressed as fold change on a log2 scale for the control and HgCl_2_-treated animals.

Exposure to inorganic mercury induced inflammation in the kidney tissue, as evidenced by a significant increase in TNF-*α* and IL-1*β* levels (*P* < 0.05, 117.08% and 172.08%, respectively). ZSCLE posttreatment substantially suppressed the kidney levels of proinflammatory cytokines compared with HgCl_2_-treated rats (Figure 7). In support of these findings, qRT-PCR results revealed that the mRNA expressions of *Tnfα* (eightfold change), *Il1β* (7-fold change), and *Nos2* (fivefold change) were significantly upregulated in the kidney of HgCl_2_-treated rats. However, ZSCLE posttreatment prevented the alteration of those genes (Figure 7).

**Figure 8:**
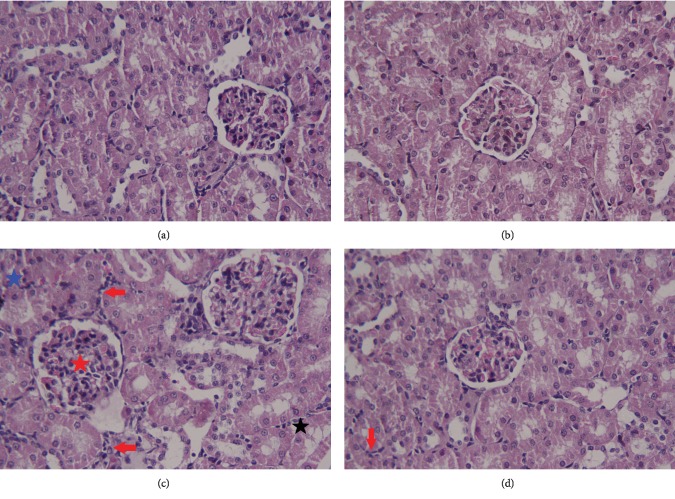
Histopathological changes in renal tissue following treatment with *Ziziphus spina-christi* leaf extract (ZSCLE) and/or mercury chloride (HgCl_2_). Kidney tissues from the control and ZSCLE-treated groups (a and b, respectively) showed normal kidney architecture. In HgCl_2_-treated rats, severe inflammation (red arrow), cytoplasmic vacuolation (black star), severe tubular necrosis and apoptosis (blue star), and congested glomeruli (red star) are shown. Posttreatment of ZSCLE (d) markedly minimized all renal lesions caused by mercury. Hematoxylin and eosin (H&E): 400x magnification.

Histopathological assessments of the control and ZSCLE-treated rats revealed the normal histology of the kidney tissue (Figures 8(a) and 8(b), respectively). In HgCl_2_-treated animals, the kidney tissue exhibited extensive damage characterized by congested glomeruli, severe infiltration of inflammatory cells, and swollen and necrotic epithelial cells (Figure 8(c)). Posttreatment of ZSCLE resulted in the preservation of normal kidney histology (Figure 8(d)).

**Figure 9:**
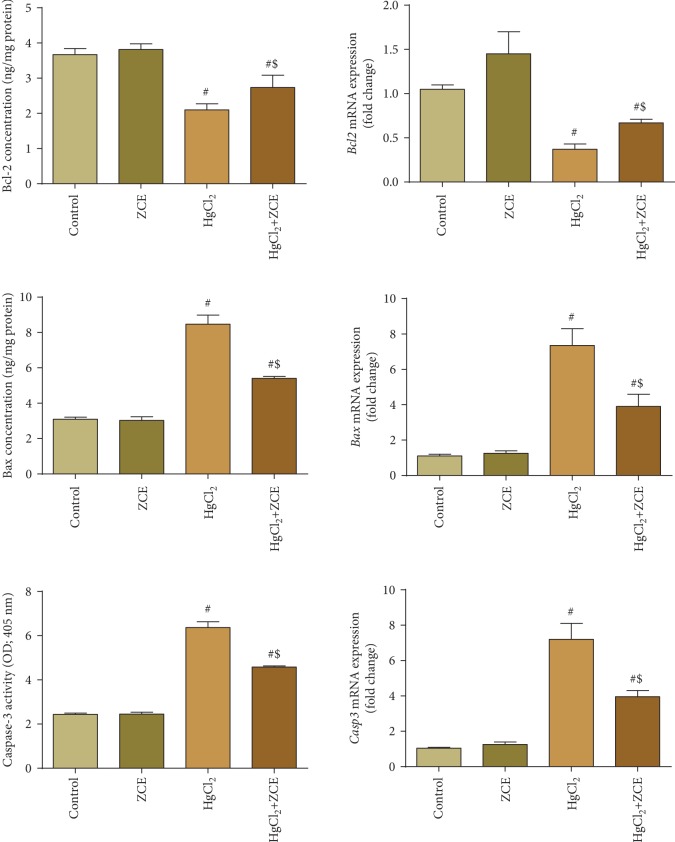
The protective effect of *Ziziphus spina-christi* leaf extract (ZSCLE) on the expression of Bax, caspase-3, and Bcl-2 in response to exposure to mercury chloride (HgCl_2_). The protein expression data are presented as the mean ± SEM (*n* = 7), with the mRNA data expressed as the mean ± SD of triplicate assays normalized to *Gapdh* and expressed as fold change on a log2 scale, for the control and HgCl_2_-treated animals.

To further explore whether HgCl_2_-induced renal injury was associated with apoptotic cell death, we conducted ELISA analysis of Bcl-2, Bax, and caspase-3 on kidney homogenates. Bax and caspase-3 protein levels were significantly increased (173.11% and 162%, respectively) in the kidney of HgCl_2_-intoxicated rats accompanied by a significant decrease in the Bcl-2 level (-42.77%). However, compared with the Hg-treated group, ZSCLE posttreatment markedly attenuated the increase in Bax and caspase-3 levels and markedly increased the Bcl-2 level. Similar to the ELISA results, qRT-PCR data revealed that *Bax* and *casp3* mRNA expressions were upregulated (sevenfold change for each) in the kidney of HgCl_2_-intoxicated rats, whereas *Bcl2* expression was downregulated (threefold change). However, Hg-induced apoptotic cell death in the kidney was suppressed by ZSCLE posttreatment (Figure 9).

## 4. Discussion

Heavy metals, including mercury, are used widely in the modern word for different activities, and their occurrence in the environment has increased tremendously. The contamination of food and water by heavy metals is known to be associated with the development of adverse reactions in animals and humans [9]. Owing to their presence in many places, heavy metals are readily absorbed by our bodies and interact with the cellular compartments, disturbing the cellular, molecular, and biochemical processes, and may lead to death [24]. Owing to its ability to absorb and excrete toxicants, the kidneys represent the major target organ of heavy metal intoxication. The magnitude of kidney damage is dependent on the nature of the toxicant, the dose, and the duration of exposure [25].

In the current study, mercury accumulation in the renal tissue was increased following HgCl_2_ administration for 28 days. Our finding is in agreement with previous investigations [26, 27], for which authors attributed this behavior to the enhancement of renal efflux transport expression, and this accumulation has been linked with the pathogenesis of renal dysfunction. The use of natural products and their active forms as chelating agents represents an effective strategy against inorganic mercury intoxication [28]. ZSCLE was able to enhance Hg clearance and decreased its accumulation in the renal tissue. We suggested that ZSCLE could be suitable for mercury chelation owing to its high flavonoid content, as flavonoids have been shown to chelate heavy metals [29]. In addition, the chelation properties of ZSCLE have been reported previously [30]. Hg accumulation in the renal tissue was associated with a decrease in the body weight and kidney index, whereas ZSCLE was able to restore those markers to near normal values, which was suggestive of its protective role against Hg intoxication.

Mercury accumulation in renal tissue has been associated with a disturbance in kidney functions, as indicated by the increase in serum levels of creatinine, urea, and Kim-1 mRNA expression. These findings are in agreement with previous studies [26, 27, 31]. Serum levels of creatinine and urea are used as markers for renal structure and function integrity. The expression of Kim-1 mRNA (*Havcr-1*) is low in normal renal tissue, while its expression is elevated after the progression of kidney injury [32]. The elevation of these renal indices has been attributed to renal tubule damage following exposure to Hg [26, 31]. ZSCLE successfully inhibited the increased serological creatinine and urea levels and Kim-1 expression induced by Hg exposure. ZSCLE was shown to restore the elevated kidney function parameters in septic mice [13]. Moreover, *Z. mauritiana* extract exerted antidiabetic effects and decreased the levels of creatinine and urea in a rat model of diabetes [33]. This effect could be attributable to the protective effect of ZSCLE against renal tubular damage as shown in the present study.

In the present work, exposure to inorganic mercury elicited a change in the balance between oxidant indices and antioxidant molecules in renal tissue, as indicated by the increase in LPO and NO production and the depletion of GSH content and GPx, GR, CAT, and SOD activities; these changes were induced in a state of oxidative stress. The decrease in the assessed antioxidant enzymes may be due to the downregulation of mRNA expression of *Gxp1*, *Gsr*, *Cat*, and *Sod2* that was recorded in the renal tissue. HgCl_2_ intoxication has been linked to the excessive generation of reactive oxygen species (ROS), including peroxide radicals, which further enhanced the peroxidation of membrane lipids [34]. The increase of NO in renal tissue may be due to the upregulation of iNOS (*Nos2*), which is the rate-limiting enzyme in NO production. NO overproduction exerts a cytotoxic role via the formation of peroxynitrite (ONOO^−^), which crosses the cell membrane, oxidizes cellular macromolecules, disturbs mitochondrial function, and enhances caspase production, resulting in cell death [35]. Moreover, Hg binds to thiol group-containing molecules, such as glutathione and glutathione-dependent antioxidant enzymes, resulting in alterations of the antioxidant detoxifying system and the development of oxidative stress in renal tissues [3]. Hg also irreversibly binds to the body's essential elements, such as zinc and selenium, leading to their elimination, which disturbs several physiological and regulatory functions, including the deactivation of the antioxidant enzymes [6]. As expected, ZSCLE significantly restrained the renal oxidative damage in response to Hg exposure via a decrease in MDA and NO production and increased GSH content and GPx, GR, SOD, and CAT activities in renal tissue. This effect was due to the upregulation of mRNA expression of antioxidant enzymes following ZSCLE treatment. ZSCLE antioxidant capacity has been reported previously [13]. ZSCLE was found to suppress the elevation of lipid peroxidation and NO production and to enhance GSH and antioxidant enzymes in cardiac and renal tissues of septic mice [13]. The authors attributed the activation of the antioxidant enzymes to the overexpression of their respective genes. In another model, *Ziziphus spina-christi* fruit extract also potentiated the content of GSH and SOD and CAT activities in hepatic tissue after carbon tetrachloride intoxication [36].

Exposure to xenobiotics including heavy metals triggers the expression of numerous genes encoding detoxifying enzymes, antioxidant proteins, and xenobiotic transporters to provide protection against oxidative challenge. This cytoprotective mechanism is mediated by the antioxidant response elements (AREs) through the activation of Nrf2 and its antioxidant response element signaling pathway including *Homx1*, *Nqo1*, *Gclm*, and *Gclc* which detoxifies and eliminates the reactive oxygen species and electrophilic molecules through conjugative reactions and enhances cellular antioxidant defense capacity [37, 38]. Hg-intoxicated rats showed downregulation of *Nfe2l2*, *Nqo1*, *Gclm*, and *Gclc*; meanwhile, *Hmox1* was upregulated in renal tissue. The depletion of *Nfe2l2* expression following treatment with inorganic mercury has been previously documented [39, 40]. Hg has been confirmed to activate Keap1, which is known to inhibit Nrf2 activity in response to xenobiotic exposure [34]. *Nfe2l2* downregulation may also explain the decrease in the assayed antioxidant molecules in the current experiment. The kidney is a metabolically active organ, and it is highly challenged with toxicants and oxidizing substances. Therefore, the activation of the Nrf2-antioxidant response pathway is necessary to maintain cellular homeostasis in response to oxidative reactions [41]. Moreover, extensive evidences demonstrate the importance of HO-1 in maintaining homeostasis during oxidative insults, and its dysregulation is associated with the destabilization of several physiological processes. The upregulated *Homx1* in the renal tissue may represent an early defense response to the initial injury produced following HgCl_2_ intoxication [42]. ZSCLE was found to upregulate *Nfe2l2*, modulate *Hmox1* expression, and provide protection against Hg-induced oxidative stress in renal tissue. Almeer et al. [43] reported that ZSCLE enhanced *Nfe2l2* and *Hmox1* expression in a rat model of ulcerative colitis. In addition, *Z. jujuba* was found to induce the overexpression of *Nfe2l2* and *Hmox1* in the liver tissue of rats treated with CCl_4_ [44].

Inflammation is an important mechanism involved in Hg-induced renal toxicity. In the current study, Hg intoxication triggered proinflammatory signaling via the increased production of TNF-*α* and IL-1*β*. In agreement with these findings, qRT-PCR results revealed that the mRNA expression of *Tnfα*, *Il1β*, and *Nos2* was significantly upregulated in the kidney of rats treated with HgCl_2_. Li et al. [45] reported an increase in the concentration of TNF-*α* and IL-1*β* in mouse renal tissue after Hg treatment. The authors demonstrated that Hg enhanced ROS production, which further activated nuclear factor kappa B and led to the oversecretion of proinflammatory cytokines. Pretreatment with ZSCLE induced anti-inflammatory activity after Hg exposure, as indicated by the decreased expression of *Tnfα*, *Il1β*, and *Nos2*. In a previous study, ZSCLE decreased the expression of the *Tnfα*, *Il1β*, and *Nos2* in different murine models through its antioxidant properties [12, 13, 46].

Exposure to inorganic mercury is associated with the progression of programmed cell death in the kidney. This was confirmed through the increased mRNA expression of Bax and caspase-3 (proapoptotic proteins) and the suppression of the expression of Bcl-2 (the antiapoptotic protein). The proapoptotic activity of Hg has been previously recorded [47]. Increased cell death induced by Hg might be due to the downregulation of Nrf2, which regulates the expression of different antioxidants responsible for ROS removal [34]. However, we found that ZSCLE treatment was able to activate this antioxidant signaling pathway in response to Hg intoxication. The antiapoptotic activity of ZSCLE may result from its ability to quench ROS, as suggested by numerous studies [43, 46, 48].

## 5. Conclusions

The current report reveals that exposure to inorganic mercury at a subchronic dose impairs renal structure and function, as evidenced by Hg accumulation, body weight loss, decreased renal indices, elevation of kidney function parameters (creatinine, urea, and Kim-1), disturbance in the balance between oxidants (lipid peroxidation and nitric oxide) and antioxidants (glutathione, superoxide dismutase, catalase, glutathione peroxidase, and glutathione reductase), suppression of the Nrf2-antioxidant response antioxidant pathway (*Nfe2l2*, *Homx1*, *Nqo1*, *Gclm*, and *Gclc*), enhancement of proinflammatory signaling through the upregulation of *Il1β*, *Tnfα*, and *Nos2* expression, and potentiation of proapoptotic activity. However, ZSCLE exerted a beneficial role against renal toxicity induced by mercury through the reversal of these alterations to near normal values. These effects were due to its chelation and antioxidant, anti-inflammatory, and antiapoptotic activities. Therefore, we suggested that ZSCLE could be used to prevent or minimize the pathological changes in the kidney induced by mercury exposure.

## Figures and Tables

**Table 1:** Primer sequences of genes analyzed by using real-time PCR.

Name	Accession number	Sense (5′-3′)	Antisense (5′-3′)
*Gapdh*	NM_017008.4	GCATCTTCTTGTGCAGTGCC	GATGGTGATGGGTTTCCCGT
*Gclc*	NM_012815.2	TGTCGCTGGGGAGTGATTTC	GGTCAGACTCGTTGGCATCA
*Gclm*	NM_017305.2	AGTGGGCACAGGTAAAACCC	CAATGCTTCCTGTGAGTGCG
*Nqo1*	NM_017000.3	ATTGTATTGGCCCACGCAGA	TCATATCCCAGGCCACCTGA
*Sod2*	NM_001270850.1	AGCTGCACCACAGCAAGCAC	TCCACCACCCTTAGGGCTCA
*Cat*	NM_012520.2	TCCGGGATCTTTTTAACGCCATTG	TCGAGCACGGTAGGGACAGTTCAC
*Gpx1*	NM_017006.2	CGGTTTCCCGTGCAATCAGT	ACACCGGGGACCAAATGATG
*Gsr*	NM_053906.2	TGCACTTCCCGGTAGGAAAC	GATCGCAACTGGGGTGAGAA
*Nfe2l2*	NM_031789.2	GGTTGCCCACATTCCCAAAC	GGCTGGGAATATCCAGGGC
*Hmox1*	NM_012580.2	GCGAAACAAGCAGAACCCA	GCTCAGGATGAGTACCTCCCA
*Havcr1*	NM_173149.2	TGGCACTGTGACATCCTCAGA	GCAACGGACATGCCAACATA
*Bcl2*	NM_016993.1	CTGGTGGACAACATCGCTCTG	GGTCTGCTGACCTCACTTGTG
*Bax*	NM_017059.2	GGCGAATTGGCGATGAACTG	ATGGTTCTGATCAGCTCGGG
*Casp3*	NM_012922.2	GAGCTTGGAACGCGAAGAAA	TAACCGGGTGCGGTAGAGTA
*Tnfα*	NM_012675.3	GGCTTTCGGAACTCACTGGA	CCCGTAGGGCGATTACAGTC
*Il1β*	NM_031512.2	GACTTCACCATGGAACCCGT	GGAGACTGCCCATTCTCGAC
*Nos2*	NM_012611.3	GTTCCTCAGGCTTGGGTCTT	TGGGGGAACACAGTAATGGC

*Gapdh*: glyceraldehyde 3-phosphate dehydrogenase; *Sod2*: manganese-dependent superoxide dismutase (MnSOD); *Cat*: catalase; *Gclc*: glutamate-cysteine ligase catalytic subunit; *Gclm*: glutamate-cysteine ligase modifier subunit; *Gpx1*: glutathione peroxidase; *Gsr*: glutathione reductase; *Nqo1*: NAD(P)H quinone dehydrogenase 1; *Nfe2l2*: nuclear factor-erythroid 2-related factor 2; *Hmox1*: heme oxygenase 1; *Havcr1* (*Kim1*): hepatitis A virus cellular receptor 1 (kidney injury molecule-1); *Bcl2*: B-cell lymphoma 2; Bax: Bcl-2-like protein 4; *Tnfα*: tumor necrosis factor alpha; *Il1β*: interleukin-1 beta; *Nos2*: nitric oxide synthase 2.

## Data Availability

All relevant data are within the paper.
